# Increased sensitivity for detecting malaria parasites in human umbilical cord blood using scaled-up DNA preparation

**DOI:** 10.1186/1475-2875-11-62

**Published:** 2012-03-05

**Authors:** Spencer D Polley, Colin J Sutherland, Fiona Regan, Maha Hassan, Peter L Chiodini

**Affiliations:** 1 Department of Clinical Parasitology, Hospital for Tropical Diseases, Mortimer Market, University College London Hospitals NHS Foundation Trust, London WC1E 6JB, UK; 2 London School of Hygiene and Tropical Medicine, Keppel Street, London WC1E 7HT, UK; 3 NHS Blood & Transplant, Colindale Avenue, London NW9 5BG, UK

## Abstract

**Background:**

All mothers donating umbilical cord blood units to the NHS cord blood bank undergo an assessment for the likelihood of prior exposure to malaria infection. Those deemed at risk due to a history of travel to, or residence in, malaria endemic regions are screened serologically to detect anti-malaria antibodies. A positive result excludes the use of the cord blood for transplant therapy unless a risk assessment can ensure that malaria transmission is extremely unlikely. This paper details the screening of cord blood units from malaria serology positive mothers to detect malaria parasite DNA using a highly sensitive nested PCR.

**Methods:**

Uninfected blood from a healthy volunteer was spiked with known quantities of malaria parasites and 5 millilitre and 200 microlitre aliquots were subjected to DNA extraction using QIAamp DNA maxi and DNA mini kits respectively. Nested PCR, to detect malarial SSU rRNA sequences, was performed on the purified DNA samples to determine the limit of detection for this assay with both extraction methodologies. Following assay validation, 54 cord blood units donated by mothers who were positive for anti-malaria antibodies were screened by this approach.

**Results:**

When DNA was purified from 5 millilitres of blood it was possible to routinely detect as few as 50 malaria parasites per millilitre using nested PCR. This equates to a significant increase in the sensitivity of the current gold standard nucleic acid amplification technique used to detect malaria parasites (routinely performed from > 200 microlitre volumes of blood). None of the 54 donated cord blood units from serology positive mothers tested positive for malaria parasites using this scaled up DNA preparation method.

**Conclusion:**

Serological testing for malaria parasites may be overly conservative, leading to unnecessary rejection of cord blood donations that lack malaria parasites and which are, therefore, safe for use in stem cell therapy.

## Background

Umbilical cord blood units (CBUs) are a rich source of stem cells. An ever increasing number of conditions have been treated using cord blood stem cells, with an estimated 20,000 people having received such therapy to date for conditions such as malignancies, immunodeficiencies [1], metabolic disorders [2], bone marrow failure [3] and haemoglobinopathies [4]. At present, for many ethnic minority patients in the UK, the likelihood of finding a suitably HLA matched donor is significantly decreased due to the predominance of Caucasian donors [5]. Furthermore, matching of ethnicity has been shown to have a significantly beneficial effect on the outcome of unrelated cord blood transplantation [2]. Due to the requirement for immune suppression with current allograft technology all CBUs must be screened (directly or indirectly) for pathogens before use. Transplacental transmission of malaria has been documented in areas which are endemic for malaria [6,7]. Those mothers donating CBUs who have visited or lived in malaria endemic countries or have had a confirmed episode of malaria in the past are therefore screened serologically using a commercially available enzyme immunoassay (Malaria Ab EIA, Lab 21, Cambridge, UK) to look for the presence of anti-malaria antibodies. A positive result excludes the use of the cord blood for transplant therapy. It is known that anti-malaria antibodies may be present long after the clearance of a malaria infection [8,9]. Although one of the antigen components (MSP2) in the assay is targeted by antibodies with a very short half life in children [10], antibodies to the other antigen component (MSP1) may be very long lived [11]. There remains the possibility, therefore, that serological testing results in the unnecessary rejection of CBUs which do not contain malaria parasites. This is especially problematic for members of ethnic minorities, given the likelihood of serology positive mothers belonging to these groups.

Currently, nested PCR [12] is the most sensitive and robust nucleic acid amplification technology (NAAT) available to screen blood for the presence of malaria parasites and, with a published sensitivity of 400 parasites per ml, is significantly more sensitive than light microscopy [13]. Current protocols generally use small volumes of blood (50-200 μl) with commercial extraction kits (e.g. QIAamp mini kit) to purify nucleic acids prior to testing by nested PCR. Using QIAamp maxi kits for isolating DNA from 5 ml of cord blood it was possible to reliably detect as few as 50 parasites per milliliter of blood in spiked blood samples. Analysis of 54 CBUs from serologically positive mothers failed to detect malaria parasites at levels above this threshold.

## Methods

Assay validation was performed with in vitro cultured parasites of the 3D7 strain of *Plasmodium falciparum*. Parasites were synchronized using sorbitol to remove all forms other than ring-stage (early) trophozoites [14]. The purified early trophozoites were washed and then re-suspended in 1 ml of fresh blood, collected in EDTA from an uninfected volunteer. This produced a 3% parasitaemia, which was determined by microscopy using thin blood films stained in Rapid Fields. Assuming a starting red cell density of 5 × 10^9 ^per ml, this equates to 1.5 × 10^8 ^parasites per ml. Serial dilutions in the same uninfected blood were made to produce samples with effective parasite densities of 5000, 500 and 50 parasites per ml. DNA (both human and parasite) was purified from 5 ml and 200 μl of frozen blood using the QIAamp DNA Blood maxi kit and the QIAamp DNA blood mini kit respectively (Qiagen, Hilden, Germany), as per manufacturer's instructions. For the 5 ml and 200 μl samples the final elution used 1 ml and 100 μl of buffer EB. From each eluate, 5 μl was analysed using a nested PCR approach as per published methods to amplify *P. falciparum, Plasmodium vivax, Plasmodium malariae *and *Plasmodium ovale *small sub-unit ribosomal RNA sequences [12]. *Plasmodium ovale *is best described as two sister species of which can be differentiated by use of the nested PCR; DNA from the so-called variant type (recently named *Plasmodium ovale wallikeri*) does not amplify with this assay [15]. To overcome this, an alternative PCR was carried out combining the first round of the nested PCR with a second round of PCR using alternative PCR primers [16]. The resultant assay amplifies both *P. ovale curtisi *and *P. ovale wallikeri *reliably. To ensure the spiked blood samples contained the correct density of parasites, the QIAamp DNA mini eluates were titred using a real-time PCR assay against DNA from 200 μl aliquots of a ten fold dilution series of the WHO international standard for *P. falciparum *[17,18]. All PCRs were carried out at the Parasite Reference Laboratory, a fully CPA accredited lab, which routinely carries out malaria diagnostics.

Following assay validation, DNA was extracted from 54 umbilical CBU donations from mothers who were positive for malaria antibodies by the EIA assay and the DNA was amplified to determine the presence or absence of *Plasmodium *species DNA as above. The study was granted ethical approval by the Research Ethics Committee (REC) of University College London Hospitals (REC reference 07/Q0505/60) and all mothers gave written informed consent that CBUs could be used for research if they were rejected for use in transplant therapies.

## Results

In validation experiments, uninfected umbilical cord blood spiked with cultured malaria parasites was routinely positive by amplification for samples with parasite densities at or above 500 parasites per millilitre. This was true for DNA extracted from either 5 ml or 200 μl of this blood. With spiked blood containing 50 parasites per millilitre, positive amplification was achieved in 85% (17/20) of PCR reactions using DNA prepared from 5 ml of blood. By contrast, only 30% (6/20) of PCR reactions showed positive amplification when using DNA prepared from a 200 μl aliquot. Thus, at this level of parasite density there is a significant improvement in the rate of detection when using the larger blood volume for DNA preparation (P < 0.001, Yates corrected Chi squared). No amplification was seen with DNA preparations from uninfected adult human peripheral blood or umbilical cord blood spiked with lower levels of parasites.

None of the 54 CBUs analysed using manual extraction of 5 ml by the maxi preparation method showed the presence of detectable parasite DNA from any of the five parasite species tested in the assay. All positive control DNAs from infected patient samples amplified correctly in these assays.

## Discussion

The use of larger blood volumes for DNA preparation offers a significant improvement in the limit of sensitivity of detection for the diagnosis of *P. falciparum *parasites using nested PCR. This can be readily explained by the density of parasite genomes in the resultant eluates. 5 ml of blood with a parasite density of 50 parasites per ml will contain approximately 250 parasites. Assuming a 100% recovery of DNA in the 1 ml eluate, then on average there will be 1.25 parasites genomes within the 5 μl of eluate assayed. When only 200 μl of blood is extracted using Qiagen miniprep kits, such a parasite density would result in at best 0.5 parasite genomes within an amplification reaction, even where 100 μl of elution buffer is used to purify DNA in order to maximize DNA concentration. Even with the multi copy target used in this assay, 0.5 parasite genomes appears to be too little DNA to support reliable amplification. In addition to this increased DNA concentration, the ability to sample a much larger volume of blood will decrease sampling variability and also serve to increase the reliability of the assay.

Within the literature there are reports of amplification from blood samples containing as few as 20 parasites per ml [16]. It is hard to envisage how such amplification could be achieved using a standard QIAamp mini DNA extraction protocol, as is in usage in many diagnostic laboratories. Extraction of 200 μl samples containing 20 parasites per ml will produce eluates containing at best only 0.04 parasites genomes per μl. Even where 5 μl of DNA is used in a PCR reaction, this would equate to only 0.12 parasite genomes per reaction and it is surprising that such a concentration could produce reliable amplification with a single round PCR. Indeed, in a recent report on different PCR methodologies using QIAamp mini DNA extraction protocol investigators were unable to amplify less than 400 parasites per ml routinely [13]. It is possible that incorrect estimation of parasite density by slide microscopy may result in the reporting of inaccurate limits of detection in the literature. Titration of DNA samples against the internal standard for *P. falciparum *using real-time amplification allows an unbiased estimation of parasite density and therefore a greater confidence of the limits of detection of a given assay. Our reported limits of detection for nested PCR using QIAamp mini kit derived DNA are also in good accordance with a comparable study [13].

Even though the extraction of 5 ml blood volumes represents a significant improvement in diagnostic sensitivity, it most likely that the time and costs involved in the manual extraction of large volumes of blood and the subsequent analysis by nested PCR would preclude such a technology for the routine diagnosis of malaria. Nor could such an approach feasibly be applied to the wide spread identification of very low level infections as part of a malaria elimination programme, as again the cost in materials and personnel-hours would be prohibitive. However, for applications such as the screening of material for transplantation, where it is essential that sensitivity is maximized to prevent the transference of parasites into an immuno-compromized person, the assay could prove a valuable method to help ensure the safety of donations from Ab-positive individuals.

From studies within locations endemic for malaria, the probability of detecting malaria parasites within CBUs has been shown to be significantly increased by a high density of parasites in the mother's peripheral blood and intervillous placental blood. Within a cohort of Kenyan women, those with peripheral blood parasite densities in the highest tercile (geometric mean of 1,226,016 parasites per ml) had a three fold higher probability of having malaria parasites in their cord blood (probability of having malaria parasites = 0.43) compared to the middle tercile (probability of malaria parasites = 0.13, geometric mean = 13,368 parasites per ml). However, a lack of detectable parasites in the peripheral blood is not refractory to the presence of parasites in the cord blood (6.5% of women who had no detectable circulating parasites had parasites in their cord blood) [7]. It is therefore necessary to screen the cord blood directly rather than maternal peripheral blood to be certain that parasites are absent from a CBU. Within this study of 54 umbilical CBU donations, even with the use of an assay with significantly increased sensitivity, we found no evidence of infection with malaria parasites. We conclude that it is highly unlikely that any of the donations had a parasite density of greater than 50 parasites per millilitre (0.05 parasites per μl). If this is generally applicable to donations from serologically positive mothers then it may be that the majority of malaria Ab-positive CBU donations received in the UK, but deemed unsuitable for transplant, actually lack malaria parasites. This unnecessarily reduces the availability of an already scarce resource. It is worth noting that the likelihood of transplacental transmission may be significantly increased in cohorts tested in malaria endemic locations (compared to the 54 CBUs within our study) due to infection with *P. falciparum *variants known to preferentially target pregnant women and establish placental infections [19,20]. However, among UK based mothers with detectable malaria antibodies, the likelihood of infection with such variants would be very low if the causative episode(s) of malaria infection occurred before pregnancy. In such cases, a negative PCR result would support the use of these units for transplant therapies as posing a very low risk for malarial transmission. However, if potential exposure to malaria parasites occurred during pregnancy a positive maternal antibody test even with a negative PCR result would require further risk assessment.

Given that this assay requires 5 ml of blood it may be unrealistic to run the assay routinely on all CBU donations from mothers potentially exposed to malaria, but as standard practice in the NHS cord blood bank is to collect blood samples for red cell grouping from red cell "waste" product removed during the processing of CBUs, an additional 5 ml sample could be taken for malaria PCR testing in the event of a positive malaria antibody result. Even where CBUs are not to be tested directly, the risk of malaria infection from serologically positive mothers needs to be re-evaluated in the light of this work. This would be especially relevant in the cases of recipients suffering from illnesses with high childhood mortality, and where prognosis has been shown to be dramatically improved following transplantation of umbilical cord blood stem cells [2].

## Conclusion

Malaria antibody positivity in a UK mother who donates cord blood should not be an automatic contraindication to its subsequent use in stem cell transplantation. Transplantation could proceed after an individual risk assessment, preferably including NAAT, with careful monitoring of the recipient.

## Competing interests

The authors declare that they have no competing interests.

## Authors' contributions

Study design by CJS, SDP, PLC, MH and FR; maternal malaria risk data collected by MH; DNA extraction and amplification was performed by SDP; Manuscript was written by SDP, CJS, FR and PLC. All authors read and approved the final manuscript.
